# Forecasting migraine with time-series machine learning from mobile health data

**DOI:** 10.1186/s10194-026-02346-7

**Published:** 2026-03-30

**Authors:** Fahim Faisal, Amalie C. Poole, Antonios Danelakis, Tore Wergeland, Marte Bjørk, Andrej N. Khanevski, Espen Saxhaug Kristoffersen, Tjaša Kumelj, Iben C. K. Larsen, Oda V. Lunder, Manjit Matharu, Parashkev Nachev, Melanie R. Simpson, Erling Tronvik, Kjersti G. Vetvik, Bendik S. Winsvold, Lise R. Øie, Ane B. Øvrevik, Anker Stubberud

**Affiliations:** 1 NorHead Norwegian Centre for Headache Research, Norwegian University of Science and Technology, Trondheim, Norway; 2 Department of Neuromedicine and Movement Sciences, NTNU Norwegian University of Science and Technology, Trondheim, Norway; 3 Department of Neurology and Neurophysiology, St. Olavs Hospital, Trondheim University Hospital, Trondheim, Norway; 4 Department of Computer Science, NTNU Norwegian University of Science and Technology, Trondheim, Norway; 5 Department of Clinical Medicine, University of Bergen, Bergen, Norway; 6 Department of Neurology, Haukeland University Hospital, Bergen, Norway; 7 Department of Neurology, Akershus University Hospital, Lørenskog, Norway; 8 Department of General Practice, Institute of Health and Society, University of Oslo, Oslo, Norway; 9 Department of Neurology, University Hospital of North Norway, Tromsø, Norway; 10 Headache and Facial Pain Group, UCL Institute of Neurology and National Hospital for Neurology and Neurosurgery, London, UK; 11 High Dimensional Neurology Group, UCL Queen Square Institute of Neurology, University College London, London, UK; 12 Department of Public Health and Nursing, Faculty of Medicine and Health Sciences, Norwegian University of Science and Technology, Trondheim, Norway; 13 Department of Research and Innovation, Division of Clinical Neuroscience, Oslo University Hospital, Oslo, Norway; 14 Department of Neurology, Oslo University Hospital, Oslo, Norway; 15 HUNT Center for Molecular and Clinical Epidemiology, Department of Public Health and Nursing, Faculty of Medicine and Health Sciences, NTNU Norwegian University of Science and Technology, Trondheim, Norway

## Abstract

**Background:**

Machine learning provides a powerful framework to model the complex patterns underlying migraine attack onset from real-world high dimensional datasets. In this study, we used machine learning to forecast headache days using mobile health (mHealth) data from a migraine biofeedback treatment app.

**Method:**

This was a machine learning analysis of data from the BioCer clinical trial (NCT05616741) evaluating app-based biofeedback for preventive treatment of episodic migraine. Participants completed three months of daily biofeedback sessions with wearables measuring trapezius muscle tension, heart rate variability, and peripheral skin temperature. Input data for the models included summary metrics from the biofeedback sessions and daily headache diary entries. The outcome of interest was the presence of a moderate-to-severe headache (defined as an intensity of 4 or higher on an 11-point scale of 0–10) on the next calendar day and the next three calendar days. The dataset was randomly split into training, validation, and test sets. Multiple standard machine learning architectures, foundation models, and time-series models were trained and optimized using the area under the receiver operating characteristics curve (AUC) as the primary scoring metric. Among these three classes of machine learning models, the best optimized model in each class identified during training was applied on the unseen test set. Permutation feature importance (PFI) was created for model explainability.

**Results:**

146 individuals, with a total of 21,550 headache days, were included in the forecasting models. For the next calendar day predictions, the top performing standard machine learning approach (decision tree) and foundation model achieved a test set AUC of 0.59 (95% CI 0.56 to 0.61) and 0.55 (95% CI 0.55 to 0.56), respectively. The best time-series model achieved a test set AUC of 0.84 (95% CI 0.82 to 0.85). For the three-calendar day forecasting window, the test set performances were 0.55 (95% CI 0.53 to 0.56), 0.55 (95% CI 0.54 to 0.57), and 0.76 (95% CI 0.74 to 0.77), respectively. The most important features were headache intensity, duration of the headache, and heart rate scores.

**Conclusion:**

Time-series machine learning models using a relatively large dataset could forecast moderate-to-severe headaches with good accuracy in patients with episodic migraine.

**Supplementary Information:**

The online version contains supplementary material available at 10.1186/s10194-026-02346-7.

## Background

Migraine is a paroxysmal disorder with a substantial degree of burden and functional impairment during attacks, and in the interictal period [1]. During attacks, individuals are often confined to bed in a dark and quiet room for several hours, and absent from work and family. However, also interictally, individuals with migraine may experience visual disturbances, light- and sound sensitivity, allodynia, vestibular disturbances and cognitive symptoms [1, 2]. Moreover, the interictal burden also includes anticipatory worry about the next painful attack and its potential impact on planned activities [2, 3]. Such psychological and behavioral consequences could independently contribute to migraine-related disability, beyond headache frequency alone [4, 5].

Accurately predicting, or forecasting, the next attack may reduce interictal burden through predictability and planning; and may facilitate pre-emptive strategies that could reduce the risk of having the attack [6, 7]. Over recent years, there has been a rising interest in the use of artificial intelligence and machine learning to forecast headaches and migraine attacks [6, 7]. These approaches can leverage the increasing availability of large multivariate, real-world data collected through smartphones, and wearable devices. Common predictors for headache forecasting are self-reported data from headache diaries, physiological measurements from wearables, and external data such as weather changes [6–10].

At present, most models only achieve modest accuracy, which makes their clinical applicability questionable. Possible explanations for the limited accuracy are small sample sizes, short observation periods, reliance on time-independent models, and insufficient modeling of inter-individual heterogeneity [6, 7]. Further developing forecasting models using large sample sizes, and complex models that can capture temporal dynamics and inter-individual difference could lead to better forecasting results. The aim of this study was to develop and evaluate machine learning models for migraine forecasting using headache diaries and wearable data.

## Method

### Study design and participants

The data for this investigation was collected in a randomized clinical trial of home-based biofeedback therapy in Norway, from January 2023 to September 2024 (BioCer). The full study protocol can be accessed here [11]. Here we present the results of an exploratory machine learning analysis utilizing data from this study.

**Fig. 1:**
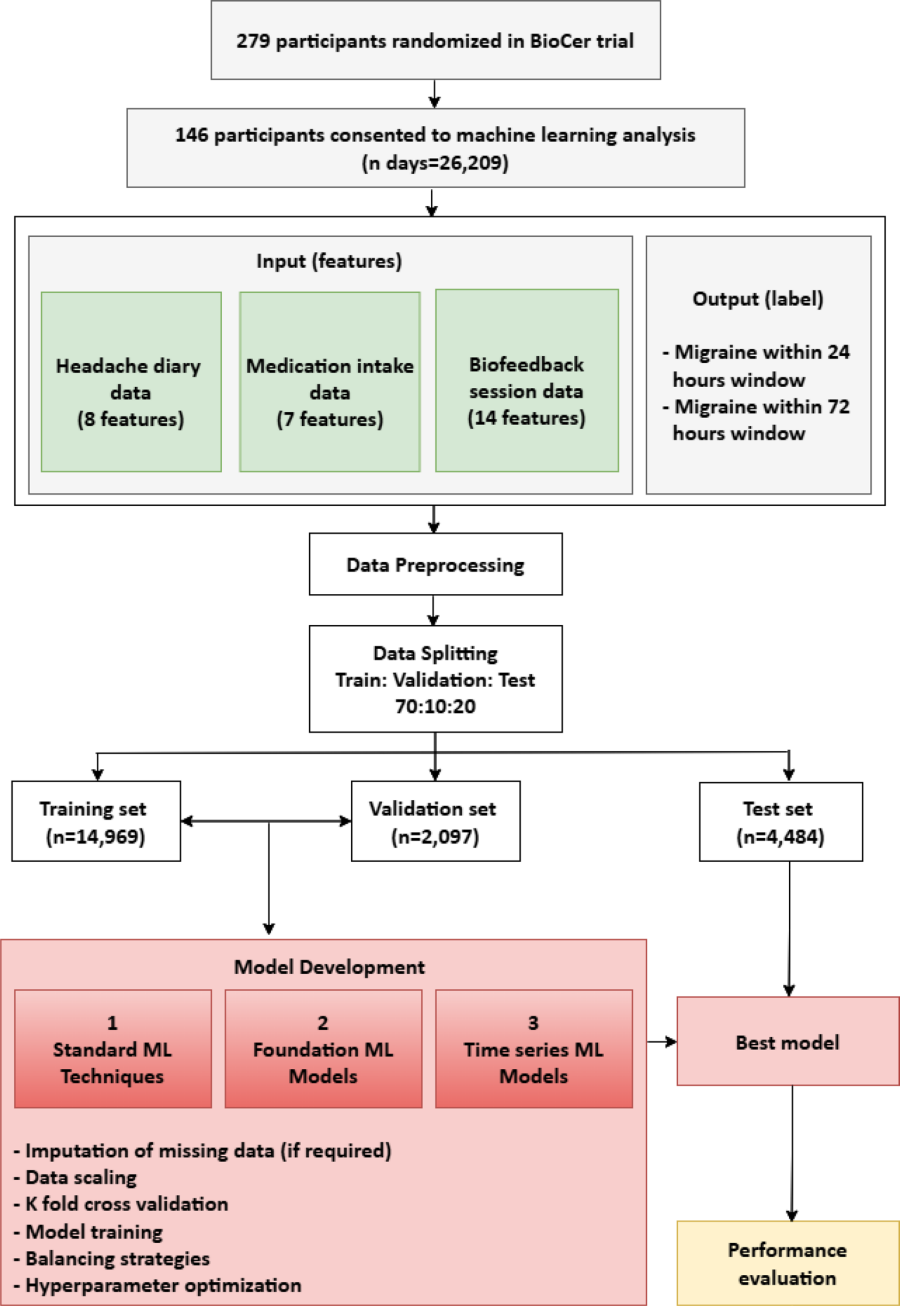
Study overview

BioCer was a clinical study to evaluate the efficacy and safety of the app-based biofeedback treatment, Cerebri, for episodic migraine in adults. The study included adults with episodic migraine with at least four monthly migraine days (see supplementary for full eligibility criteria). Participants were randomized in a 1:1 ratio to 12 weeks of treatment with Cerebri biofeedback or a wait-list control. Those assigned to wait-list were offered Cerebri biofeedback for 12 weeks in an extension phase. Participants assigned to Cerebri were asked to complete a headache diary entry and at least one 10-minute biofeedback session daily. During the biofeedback session, one sensor was attached to the skin above the trapezius muscle to measure neck muscle tension, and one sensor was attached to the index finger to measure temperature and heart rate variability. Participants randomized in the BioCer trial were invited to contribute their data collected from the headache diaries and biofeedback session to this machine learning analysis. Those who consented were included in the present study. The overall study design is demonstrated in Fig. 1.

### Data management

Data from the forecasting models came from two sources: the daily headache diary registrations, and the measurements from the biofeedback sessions. In the headache diary, participants registered the presence of a headache, its intensity, its duration, the use of acute medications and menstruation, if applicable. Acute medications were categorized into seven groups by mechanisms of action: Paracetamol, NSAIDs, Triptans, Antiemetics, Opioids, calcitonin gene related peptide (CGRP) receptor antagonists, and others. We also recorded the weekday and the calendar month. For the biofeedback session data, we calculated the session-wise maximum and minimum heartrate, temperature, and surface electromyographic-voltage during each biofeedback session. The mean heartrate, mean temperature, and the root mean voltage for each session as well as the heart rate variability pulse wave in the high and low frequency domain were calculated. Because participants were allowed to complete more than one biofeedback session daily, we used the completed sessions over interrupted or short sessions. If there were multiple complete sessions per day, we used data from the first session and then recorded as an additional variable the number of sessions that day. Because biofeedback sessions were not necessarily completed every day, the biofeedback features for days with missing biofeedback data were set to a placeholder value of − 1. After these preprocessing steps, only two features (“headache duration” and “breathing pacer”) had missing data. This missingness was considered missing completely at random and imputed with a modified implementation of the MICE (multivariate imputation by chained equation) concept [12] which provides a single and robust imputation. The full list of features is provided in the online Supplementary Material.

The dataset was split in a randomized manner into training, validation, and test sets, in a 7:1:2 ratio, stratified on the outcome. To avoid data leakage, the data was split so that no participant had data in more than one of the training, validation, or test sets. Partitions were kept separated during training. Models were evaluated without scaling, with min-max scaling, and with standard scaling. Class imbalance was addressed with multiple balancing strategies (see below).

### Outcomes

The primary outcome was defined as the presence of any moderate-to-severe headache—rated on an 11-point 0–10 numeric rating scale—in the calendar day following a headache diary entry and/or biofeedback session. The secondary outcome was defined as the presence of any moderate-to-severe headache in at least one of the three calendar days following a valid headache diary entry. Instances with missing diary entries on the following or any of the three following days, respectively, were therefore excluded from the analysis.

### Predictive modelling

Three main categories of forecasting models were trained and evaluated. First, we evaluated standard machine learning architectures including logistic regression, support vector classifiers, stochastic gradient descent, naive bayes, decision tree classifiers, random forest classifiers, gradient boosting machines, adaptive boosting, extreme gradient boosting, gaussian process, extra tree, bagging classifier, discriminant analysis, LightGBM classifier, k Nearest Neighbors classifier. Second, the foundation model Tabular Prior data Fitted Network (TabPFN) was evaluated. It is a machine learning model for tabular datasets which uses transformer architecture. It is intended for supervised classification and regression analysis on small- to medium-sized datasets, e.g., up to 10,000 samples. As our dataset exceeded 10,000 samples, we batched our data into six chunks for it to enable processing. Third, we explored several time series machine learning models including autoregressive integrated moving average (ARIMA), seasonal autoregressive integrated moving average (SARIMA), recurrent neural networks (RNNs), long short-term memory (LSTM), gate recurrent unit (GRU) and gate recurrent unit – decay (GRU-D) models. In GRU-D, we used a sliding window approach where the model was trained on a fixed-length sequence of recent observations (e.g., days 1–6) to predict the next point (day 7), then shifting the window forward (days 2–7 to predict day 8), enabling dynamic learning from the most recent data while preserving temporal dependencies. Headache outcomes were defined per calendar day. Multi-day headache episodes were not collapsed into single events; instead, each day with a reported headache was treated as a separate label in the prediction task. To address potential inflation of performance due to prediction of headache continuation (i.e. multiday headaches), we performed two sensitivity analyses. In the first, we excluded information on present day headaches (presence of headache, its intensity and duration). In the second, we predicted moderate-to-severe headaches after a headache-free calendar day. These analyses would help better separate attack persistence from true attack onset prediction.

Models were trained on the training dataset and evaluated throughout the model development process by ten-fold cross validation on the training set and fine tuning according to performance on the validation dataset. For the best candidate models, hyperparameters were optimized with a Randomized search with 100 iterations and thereafter a Grid search strategy within the promising parameter ranges identified in the randomized search. Moreover, several feature selection strategies were explored including ANOVA which is best for linear separability, mutual information strategies (MIS) which capture nonlinear relationships and random forest-based (RF) feature importance ranking for complex nonlinear relationships and interactions. We also tweaked the minimum threshold for considering a feature to be important and combined all the techniques to see if that would make any impact to improve predictability. As balancing strategies, typical randomized undersampling, oversampling and SMOTE were implemented. Forest diffusion models were also implemented to generate new data and balance minority samples. We explored generating 20%, 40%, 60% and 80% minority data samples and explored how this data generation and resampling affected performance in the training and validation.

The area under the receiver operating characteristics curve (AUC) was used as the primary scoring metric. We also calculated balanced accuracy, sensitivity, and specificity. The top performing model was decided based on a combination of cross-validated and validation set performance. The top performing model for each of the three classes was applied to the held-out corresponding test set to quantify out of sample performance. Data was reported as means, standard deviations (SD) for the train sets, and bootstrapping with 1000 iterations was used to derive 95% confidence intervals (CI) for the test set performance parameters.

### Model explainability

For the best performing model, Permutation Feature Importance (PFI) was used to evaluate the importance of each input variable. PFI measures feature importance by randomly shuffling the values of a feature and observing the resulting decrease in the model’s predictive performance, thereby quantifying how much the model relies on that feature for accurate predictions. All statistical analyses were performed, and figures were made using Python v3.10 (Python Software Foundation) with the open-source packages (see supplementary for full software package information).

## Result

### Participants and demographics

Two hundred and seventy-nine patients were randomized in the BioCer study. Among them, 146 patients consented to participate in the machine learning analysis and completed a total of 26,209 headache diary entries and 9,678 biofeedback sessions. 4,659 days were not followed by a headache diary entry. A total of 21,550 days were included for the next calendar day predictions. There were 14,969 days in the training set, 2,097 days in the validation set, and 4,484 days in the test set. The dataset was imbalanced with 17,489 headache free days and 4,061 moderate-to-severe headache days. A total of 25,890 days were included in the three-calendar day forecasting window, with 18,047, 2,539, and 5,304 days for train, validation and test sets respectively; and 16,006 headache free periods and 9,884 periods with moderate-to-severe headaches. Patient demographics are summarized in Table 1.

**Table 1:** Subject characteristics and baseline demographics

Sex, female, *n* (%)	Total Participants (*n* = 146)
	132 (90.41)
Age, years, mean (SD)	40.83 (10.74)
Height, cm, mean (SD)	167.13 (15.02)
Weight, kg, mean (SD)	72.99 (15.18)
Paid work, n (%)	124 (84.93)
Migraine with aura, n (%)	4 (2.73)
Migraine without aura, n (%)	80 (54.78)
Migraine with and without aura, n (%)	62 (42.49)
Monthly migraine days in baseline (SD)	7.08 (2.04)
Monthly headache days in baseline (SD)	8.81 (2.74)
Use of preventive medication, n (%)	65 (44.5)
Not using preventive medication, n (%)	81 (55.5)
>= 1 comorbidity, n (%)	130 (89.04)
No comorbidity, n (%)	16 (10.96)
Currently student, n (%)	18 (12.32)
Educational level completed, n (%)	
- Primary school	3 (2.02)
- High school	13 (8.91)
- Technical college	10 (6.84)
- Tertiary education (3 years or less	53 (36.33)
- Tertiary education (more than 3 years)	63 (43.09)

SD = standard deviation

### Predictive modelling performance

**Fig. 2:**
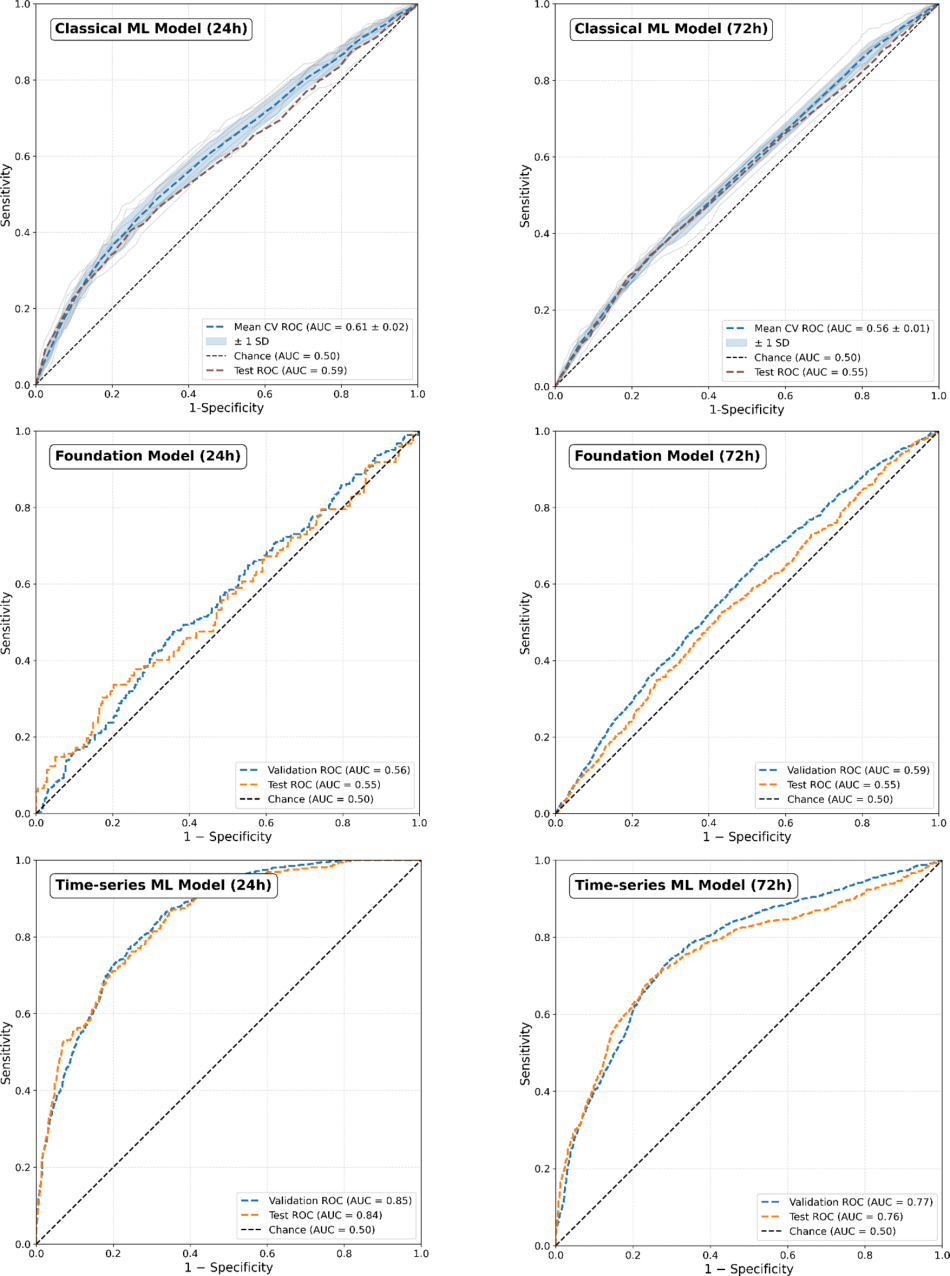
Receiver operating characteristics (ROC) curve with training and test set performance for the top performing classical machine learning model, foundation model and time series model for the next calendar day predictions, and next three-calendar day forecasting windows. For classical ML algorithms, the ten-fold cross validation with standard deviation (SD) is depicted as the blue line (mean) and shaded gray area (± 1SD). The dark maroon line represents the test set ROC curve. The test set performance was faithful to the training set. The dotted black line represents the area under the curve (AUC) for classification by chance

Feature selection strategies, scaling and resampling, including forest diffusion, did not result in any increased performance in the training or validation sets. Therefore, all features were included in the models, the data was not scaled, and no resampling was performed. Among all the data, there were a total of 4 missing data points for the features “headache duration” and “breathing pacer”. These were imputed with MICE. The best standard 24-hour machine learning model was the decision tree classifier with a mean cross-validated AUC of 0.61 (SD = 0.02) and a held-out test set AUC of 0.59 (95% CI 0.56 to 0.61) (Fig. 2). Correspondingly, TabPFN achieved a mean cross-validated AUC of 0.56 (SD = 0.03) and a held-out test AUC of 0.55 (95% CI 0.55 to 0.56). The best time-series model was GRU-D and it achieved a mean cross validated AUC of 0.85 (SD = 0.02) with a held-out test set AUC of 0.84 (95% CI 0.82 to 0.85). For the standard 72-hour machine learning model, the decision tree classifier performed best with a mean cross-validated AUC of 0.55 (SD = 0.01) and a held-out test set AUC of 0.55 (95% CI 0.55 to 0.56). TabPFN achieved a mean cross-validated AUC of 0.59 (SD = 0.02) and a held-out test AUC of 0.55 (95% CI 0.54 to 0.57). The best time-series model was GRU-D and it provided a mean cross validated AUC of 0.77 (SD = 0.02) with a held-out test set AUC of 0.76 (95% CI 0.74 to 0.77). The results of the best models are tabulated in Table 2.

**Table 2:** Predictive model results

	Performance Metrics	24 h train (SD)	24 h test (95% CI)	72 h train (SD)	72 h test (95% CI)
Classical ML Models (decision tree classifier)	AUC	0.61 (0.02)	0.59 (0.56–0.61)	0.55 (0.01)	0.55 (0.53–0.56)
	Accuracy	0.58 (0.02)	0.58 (0.56–0.60)	0.54 (0.01)	0.54 (0.52–0.55)
	Sensitivity	0.44 (0.05)	0.49 (0.45–0.52)	0.40 (0.07)	0.37 (0.34–0.38)
	Specificity	0.72 (0.01)	0.66 (0.64–0.67)	0.68 (0.08)	0.70 (0.69–0.71)
Foundation Model (TabPFN)	AUC	0.56 (0.03)	0.55 (0.55–0.56)	0.59 (0.02)	0.55 (0.54–0.57)
	Accuracy	0.55 (0.04)	0.54 (0.52–0.55)	0.56 (0.03)	0.54 (0.53–0.55)
	Sensitivity	0.42 (0.06)	0.36 (0.34–0.39)	0.45 (0.02)	0.35 (0.34–0.36)
	Specificity	0.67 (0.02)	0.75 (0.73–0.76)	0.66 (0.06)	0.72 (0.71–0.72)
Time-series ML Models (GRU-D)	AUC	0.85 (0.02)	0.84 (0.82–0.85)	0.77 (0.02)	0.76 (0.74–0.77)
	Accuracy	0.66 (0.01)	0.67 (0.67–0.68)	0.71 (0.01)	0.70 (0.70–0.71)
	Sensitivity	0.38 (0.01)	0.39 (0.36–0.39)	0.64 (0.03)	0.58 (0.57–0.61)
	Specificity	0.94 (0.08)	0.95 (0.95–0.96)	0.78 (0.08)	0.83 (0.82–0.85)

For the training set, the mean of each fold ± 1SD is reported. For the test set, 95% CI is reported

SD = standard deviation, CI = confidence interval, TabPFN = tabular prior-data fitted network, GRU-D = decayed gate recurrent unit

In the sensitivity analysis excluding same-day headache presence/intensity/duration, GRU-D provided a mean cross validated AUC of 0.84 (SD = 0.02) with a held-out test set AUC of 0.83 (95% CI 0.82 to 0.84). In the sensitivity analysis predicting a new onset moderate-to-severe headache after a headache-free calendar day, the sample size for the train, validate and test were 8,654, 1,134 and 2,433, respectively. GRU-D resulted in a mean cross validated AUC of 0.75 (SD = 0.02) with a held-out test set AUC of 0.76 (95% CI 0.74 to 0.78). The complete results of the sensitivity analyses are tabulated in the online Supplementary Material.

### Model explainability

**Fig. 3:**
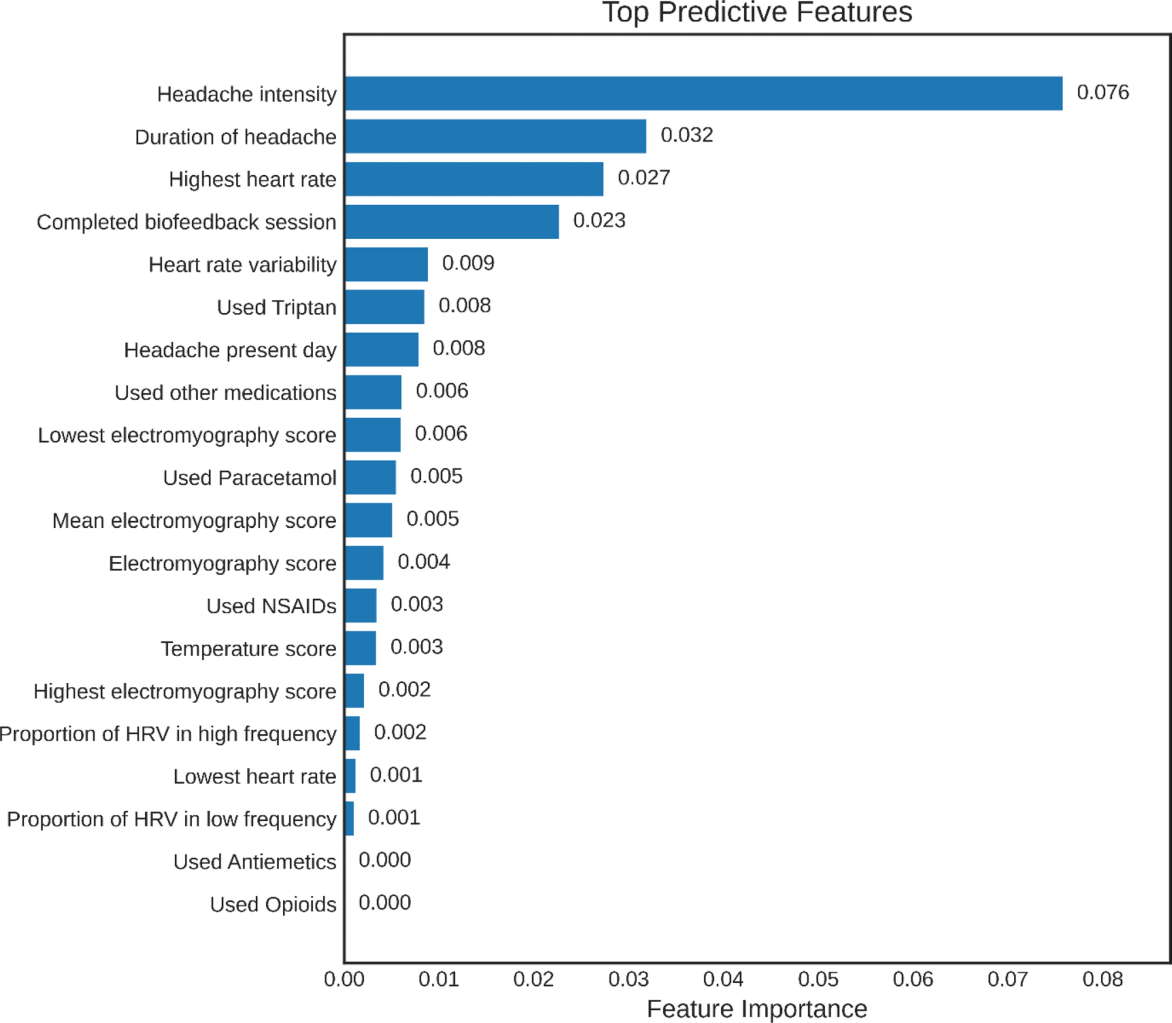
Permutation Feature Importance (PFI) of the 20 most predictive features in the best-performing time-series model for forecasting moderate-to-severe headache the next calendar day. Importance is defined as the decrease in model performance after randomly permuting each feature, thereby disrupting its association with the outcome. Features are ordered from most to least important. Headache intensity, headache duration, and maximum heart rate demonstrate the strongest contribution to predictive performance, while other behavioral and physiological variables contribute more modestly. A complete description of all features is provided in the Supplementary Material

Figure 3 shows the PFI plot indicating that the most important features were headache intensity, headache duration, and heart rate scores during biofeedback sessions.

## Discussion

### Principal findings

In this study, we developed and evaluated what we believe to be one of the most accurate migraine forecasting models to date. While the conventional machine learning approaches and the foundation models only performed modestly, the neural network-based time-series GRU-D model achieved an out-of-sample AUC of 0.84 for forecasting moderate-to-severe headache the next calendar day and 0.76 for forecasting within three calendar days.

### Interpretation

Two recent reviews of machine learning headache forecasting models have only identified a handful of published studies so far [6, 7, 13]. Siirtola et al. used headache diary data and sleep measurements from seven individuals with migraine to predict migraine attacks [14]. The per-person accuracy was good, but the user-independent accuracy was only 47.4%. Katuski et al. linked headache diary data with weather data and achieved a R^2^ of 53.7% for predicting headache occurrence in a test set [9]. In a pilot study of the same biofeedback setup used in the present study, the test-set AUC was 0.62. Houle et al. used self-reported stress and headache diary data for forecasting headache with an AUC of 0.65 [15]. The best forecasting model in our study achieved an out-of-sample AUC of 0.84. However, this relatively high AUC is constituted of a low sensitivity (0.39) and a high specificity (0.95), indicating that the model excels at identifying headache-free days at the cost of poorly predicting headache days. On the other hand, the best model for forecasting within three calendar days was more balanced with a sensitivity of 0.58, a specificity of 0.83, and an AUC of 0.76. In forecasting models, the question remains as to whether specificity or sensitivity should be prioritized. Will patients have most benefits from knowing when they will have a headache, or knowing when they will not have a headache? Arguably, sensitivity is more important than specificity because it enables pre-emptive measures and planning. This is especially true for less frequent episodic migraine, where identifying one of the few painful days makes more sense than always predicting the default, i.e. no headache. On the contrary, a high specificity means that there are few “false alarms” which could unnecessarily prohibit participation in work and social activities in fear of having a migraine. Nevertheless, the low sensitivity of the model at current likely limits its clinical utility.

Evidently from the available literature, migraine forecasting seems to be an inherently difficult task. As previous works have pointed out, models seem to be limited by small sample sizes, uncertainty about appropriateness of model selection, and uncertainty about the feature space to be included in the models [6, 7]. The accuracy and generalizability of machine learning is directly correlated with the amount of available data, and performance increases with increasing input data [16–18]. When it comes to model selection, studies published so far primarily use standard machine learning architectures which likely fail to capture important temporal dynamics. The architectures that model temporal dependencies may better capture preictal dynamics that are important for forecasting [19, 20]. Indeed, our results indicate that time-series models significantly outperform the other approaches. We included six days of observations to predict the seventh day. This timeframe was selected to capture short-term temporal dynamics that may precede the onset of moderate-to-severe headache episodes. Physiological and behavioral changes associated with migraine, including alterations in autonomic function, muscle tension, sleep patterns, and stress levels, may develop several days before an attack. Therefore, incorporating approximately one week of historical observations allows the model to capture potential prodromal patterns that may contribute to migraine onset. At the same time, this window length represents a practical compromise between capturing sufficient temporal information and maintaining adequate data availability across participants, as longer windows may increase the number of non-registered days and reduce the number of usable sequences. While time-series modelling seems to work well for temporal data, such as in this study, it might be less suited for cases with irregular sampling, substantial missing data, or limited longitudinal observations per patient.

The optimal feature space for migraine forecasting models is unknown, likely owing to the complex neurobiological processes that underlie the migraine cycle and migraine attack initiation [21]. The migraine prodrome involves hypothalamic dysregulation, autonomic imbalance, and sensory hypersensitivity, yet these processes are neither uniform nor consistently detectable [10, 22]. Hormonal changes, external stimuli and other patient-level factors may all contribute to the attack initiation [23]. The migraine prodrome presents a wide range of symptoms such as fatigue, neck stiffness, food cravings, and mood changes which could be potential predictors. However, their prevalence and timing are inconsistent, with some patients experiencing multiple symptoms and others none at all [24]. Autonomic nervous system (ANS) alterations, potentially captured by wearables, have also been proposed as biomarkers for migraine forecasting. Still, evidence suggests that autonomic symptoms do not consistently correlate with attack frequency or severity [25]. Moreover, ANS dysfunction exhibits bidirectional patterns—sympathetic hypoactivity interictally and hyperactivity ictally—making it difficult to define stable predictive markers [26].

Our approach seems to overcome the first two of the above-mentioned challenges, i.e. the sample size is larger, and the best models are flexible, and time-dependent, potentially explaining why the performance is superior to previous publications. Still, the latter task of identifying the optimal feature space for forecasting remains. Even more so, the means to non-invasively and effectively capture this information needs to be explored. Can for example central nervous and hormonal changes be captured indirectly through wearables measuring simple physiological data, or registration of premonitory epiphenomena and symptoms? Future works should focus on which types of data are most important for forecasting models, and how this data may best be captured in tools that can be made available to patients.

Another important topic for discussion is how access to forecasting information affects patients, and whether reliable migraine prediction reduces migraine burden and improves quality of life. Though forecasting may help with planning and pre-emptive strategies, it could also pose problems. For instance, it could cause anxiety for some patients when they receive a prediction of an impending migraine. Therefore, there is a need to use prediction models cautiously, and it is recommended that there is clinical guidance in helping patients understand the prediction as probabilistic rather than certain outcomes. Moreover, although time-series models may improve headache attack prediction by capturing temporal patterns in physiological and behavioral data, whether they improve AUC depends on the quality and consistency of longitudinal observations. With sparse, irregular, or short time series, their advantage over conventional machine learning models can be limited.

Finally, it is important to state that any machine learning model should be evaluated in independent samples to ensure their generalizability and clinical value. Validation using datasets from e.g. other institutions and different clinical environments is necessary to assess its overall generalizability. Such validations must be completed before the model can be used in clinical practice. Moreover, forecasting models are likely subject to stringent regulations as a medical device which requires comprehensive assessments and approvals [27–30].

### Strengths and limitations

The most important strengths of this work are the relatively large sample size, the comprehensive machine learning approach, and strict train set test set split. However, several limitations must be acknowledged. First, despite prospective data capture, recall bias may still affect the accuracy of self-reported symptoms, particularly when patient’s complete diary entries retrospectively. Second, missing data requires imputation, which may introduce uncertainty and potentially influence model performance. Third, the dataset was derived from a biofeedback RCT population with high motivation, high adherence to daily app use, and engagement in self-management. This may introduce selection bias and not reflect real-world general migraine populations. In addition, biofeedback training may also influence physiological signals or headache patterns, which could affect forecasting performance. These factors should be considered when interpreting the results and assessing generalizability. Finally, triggers and premonitory symptoms were not collected as part of the headache diary in the BioCer trial, and thus not available for inclusion in our analysis. Inclusion of such data could have improved performance.

## Conclusion

In this study, we used simple headache diaries and wearable data to forecast moderate-to-severe headache days in individuals with episodic migraines with relatively good overall accuracy but low sensitivity. The findings indicate that sufficient sample sizes and choice of forecasting models are crucial for headache forecasting. Further work should identify the optimal features to be used for forecasting and evaluate how forecasting affects patients.

## Electronic supplementary material

Below is the link to the electronic supplementary material.

Supplementary Material 1

## Data Availability

The minimum dataset required to replicate this work contains personal sensitive information and is not publicly available nor available upon request. The analytical code may be provided upon reasonable request.
