# Supplementary material for: Forecasting migraine with time-series machine learning from mobile health data
Source: J Headache Pain. 2026 Mar 30;27(1):91. doi: 10.1186/s10194-026-02346-7 (PMC13036945; doi:10.1186/s10194-026-02346-7)
Supplement: Supplementary file 1 — Supplementary Material 1 [file 10194_2026_2346_MOESM1_ESM.docx]

**Supplementary materials**:

**Inclusion Criteria:**

- 18 years of age inclusive or older, at the time of signing the informed consent.
- Episodic migraines< with or without aura diagnosed by a neurologist/physician per International Classification of Headache Disorders 3rd edition.
- History of at least 4 and up to 14 days of migraines per 28-day period in the 3 months prior to screening (as recalled by the subject). This frequency must be confirmed in the headache diary before randomization to treatment or wait-list control.
- At least three months of experience with smartphone and access to an iOS or Android phone at home.
- Capable of giving signed informed consent which includes compliance with the requirements and restrictions listed in the informed consent form.
- Onset of migraine before age 50 years.

**Exclusion Criteria:**

- More than 14 days of headache (all types) per 28-day period.
- Subjects diagnosed with trigeminal autonomic cephalalgias and neuralgias.
- Subjects with secondary headache conditions.
- Subject with pathologies that inhibit use of the device according to the instructions for use (e.g., blindness, deafness).
- Use of non-pharmacological preventive treatment (meditation, physical therapy, psychotherapy as a headache treatment, acupuncture, etc.), with the exception of stable treatment for other indications than migraine
- Use of concurrent migraine preventive medication, with the exception of stable dose (≥3 months) monotherapy of migraine preventive medication. Subjects who have previously attempted three or more prophylactic pharmacological treatments in adequate doses, without significant clinical effect.
- Subjects taking opioids (>3 days per month) or barbiturates at the time of screening.
- Subject participates in another clinical investigation or has participated in CER-MIG-1.^1^
- Alcohol overuse or illicit drug use.
- Subject who is unlikely to follow Clinical Investigation Plan or where treatment seems futile in the opinion of the Investigator or have demonstrated an inability to sufficiently adhere to headache diary entries (<70%).

Python open-source packages:

matplotlib 3.6.1; numpy 1.23.4; pandas 1.5.0; scikit-learn 1.1.2; scikit optimize 0.9.0; seaborn 0.12.0 and shap 0.41.0.

List of features:

| **Feature type** | **Feature name** | **Description of the feature** |
| --- | --- | --- |
| **Headache features** | Self-reported headache | Headache present day |
|  | Intensity | Headache Intensity |
|  | Duration | Duration of headaches |
|  | Menstruation | Menstruation |
|  | day | Day of the month |
|  | month | Month of the year |
|  | is_weekend | Weekend |
|  | day_of_week | Day of the week |
| **Medication features** | Paracetamol | Used paracetamol |
|  | NSAIDs | Used NSAIDS |
|  | Triptans | Used triptans |
|  | Antiemetics | Used antiemetics |
|  | Opioids | Used opioids |
|  | CGRP-inhibitors | Used CGRP-inhibitors |
|  | Others | Used other medications |
| **Session features** | hrv_score | Heart rate variability |
|  | hr_max | Highest heart rate |
|  | hr_min | Lowet heart rate |
|  | emg_min | Lowest electromyography voltage |
|  | emg_max | Highest electromyography voltage |
|  | emg mean | Mean electromyography voltage |
|  | emg_score | Electromyography score |
|  | temp_min | Lowest temperature |
|  | temp_max | Highest temperature |
|  | temp_mean | Mean temperature |
|  | temp_score | Temperature score |
|  | hrv_hf_nu | Proportion of HRV in high frequency |
|  | hrv_lf_nu | Proportion of HRV in low frequency |
|  | breathing_­pacer | Breathing pacer used |

Sensitivity analysis:

|  |  |  | **Performance Metrics** | **24h train (SD)** | **24h test**  **(95% CI)** |
| --- | --- | --- | --- | --- | --- |
| **Time series ML GRU-D Model** | | | **AUC** | 0.85 (0.02) | 0.84 (0.82-0.85) |
|  |  |  | **Accuracy** | 0.66 (0.01) | 0.67 (0.67-0.68) |
|  |  |  | **Sensitivity** | 0.38 (0.01) | 0.39 (0.36-0.39) |
|  |  |  | **Specificity** | 0.94 (0.08) | 0.95 (0.95-0.96) |
| **Excluding same-day headache presence/intensity/duration** | | | **AUC** | 0.84 (0.02) | 0.83 (0.82-0.84) |
|  |  |  | **Accuracy** | 0.67 (0.01) | 0.66 (0.65-0.67) |
|  |  |  | **Sensitivity** | 0.42 (0.01) | 0.40 (0.39-0.40) |
|  |  |  | **Specificity** | 0.92 (0.08) | 0.93 (0.93-0.94) |
| **Prediction for a new-onset attack after ≥ 24h headache-free period** | | | **AUC** | 0.75 (0.02) | 0.76 (0.74-0.78) |
|  |  |  | **Accuracy** | 0.62 (0.01) | 0.61 (0.57-0.62) |
|  |  |  | **Sensitivity** | 0.39 (0.01) | 0.32 (0.29-0.33) |
|  |  |  | **Specificity** | 0.84 (0.08) | 0.88 (0.85-0.86) |
